# Superior Vena Cava Obstruction: A Rare Cause of Recurrent Esophageal Variceal Bleeding

**DOI:** 10.7759/cureus.2226

**Published:** 2018-02-26

**Authors:** Agazi Gebreselassie, Ahmad Awan, Hamid Yaqoob, Adeyinka Laiyemo

**Affiliations:** 1 Gastroenterology, Howard University Hospital; 2 Department of Internal Medicine, Howard University Hospital; 3 Department of Medicine, Howard University Hospital

**Keywords:** esophageal varices, downhill varices, superior vena cava obstruction

## Abstract

“Downhill” esophageal varices are formed in upper two-thirds of the esophagus as a consequence of a superior vena cava obstruction. We present a case of 55-year-old African-American female with a medical history of multiple comorbidities, including end-stage renal disease, who presented with an upper gastrointestinal bleed and was found to have distended neck veins on physical examination. She gave a history of the insertion of an intravenous central line in her neck area for hemodialysis purposes about six years previously. An endoscopy showed the presence of esophageal varices and computed tomography (CT) of the abdomen showed the presence of a superior vena cava (SVC) obstruction. The patient was managed supportively. This case represents a rare cause of acute upper gastrointestinal bleeding in an individual with a central line for dialysis leading to SVC thrombosis.

## Introduction

The most common cause of esophageal varices is portal hypertension. These varices are also known as ‘uphill esophageal varices’, as they are found upstream to the venous flow. These varices are usually found in the lower third of the esophagus. Conversely, ‘downhill’ esophageal varices are formed in the upper two-thirds of the esophagus as a consequence of superior vena cava (SVC) obstruction and are considered to be among the rare causes of gastrointestinal bleeding [1-2]. Non-bleeding varices may be found in patients with extrinsic or intrinsic SVC obstruction undergoing a screening upper endoscopy [1]. We present a rare case of recurrent upper gastrointestinal bleeding from a chronic SVC thrombosis in a patient with end-stage renal disease on hemodialysis.

## Case presentation

**Figure 1:**
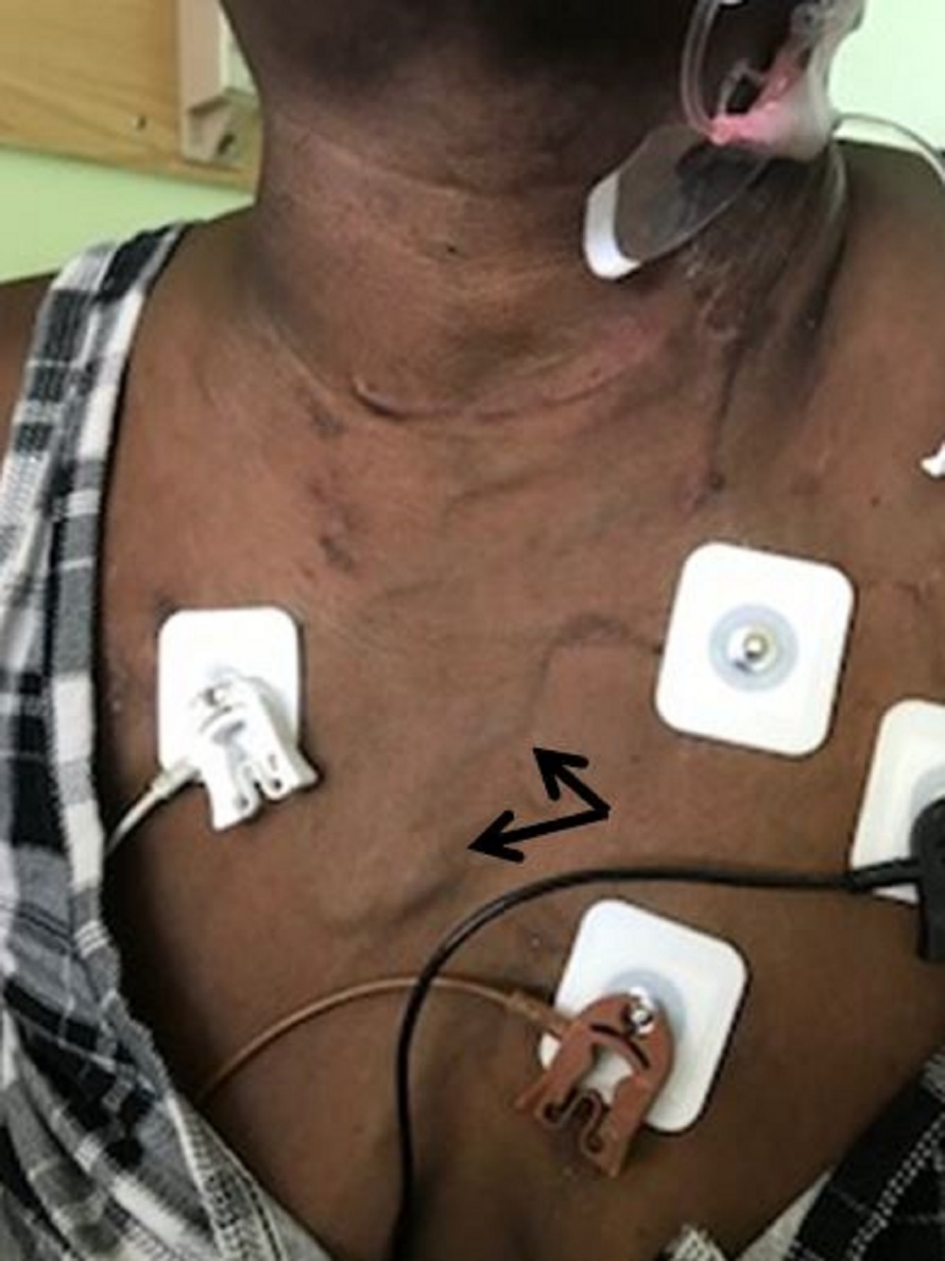
Markedly engorged neck veins (arrows)

A 55-year-old African-American female with a medical history significant for hypertension, end-stage renal disease, hypothyroidism, and history of coronary artery disease with coronary stent placement presented to the emergency department with abdominal pain and persistent vomiting of ingested material for one day. While in the emergency department, she was noted to have three episodes of painless vomiting of bright red blood. The amount of bleeding was 400 milliliters (ml), 700 ml, and 500 ml, respectively. She remembered that she had similar episodes of vomiting of blood in the past and she was told she had a bleeding vessel in her ‘food pipe’. She did not remember if any interventions were done to treat it. She gave a history of the insertion of an intravenous central line in her neck area for hemodialysis purposes about six years previously. The line was removed later on because it formed a clot. She had no history of liver disease, abdominal distension, or jaundice. On examination, she was not in acute distress. There was no conjunctival pallor and no scleral icterus. Physical examination was notable for engorged veins of the anterior chest (Figure 1). She had no ascites or other stigmata of chronic liver disease. Laboratory investigation revealed hemoglobin of 12.9 g/dL with a platelet count of 206,000 per microliter. Liver enzymes were within normal limit and liver synthetic function was preserved.

**Figure 2:**
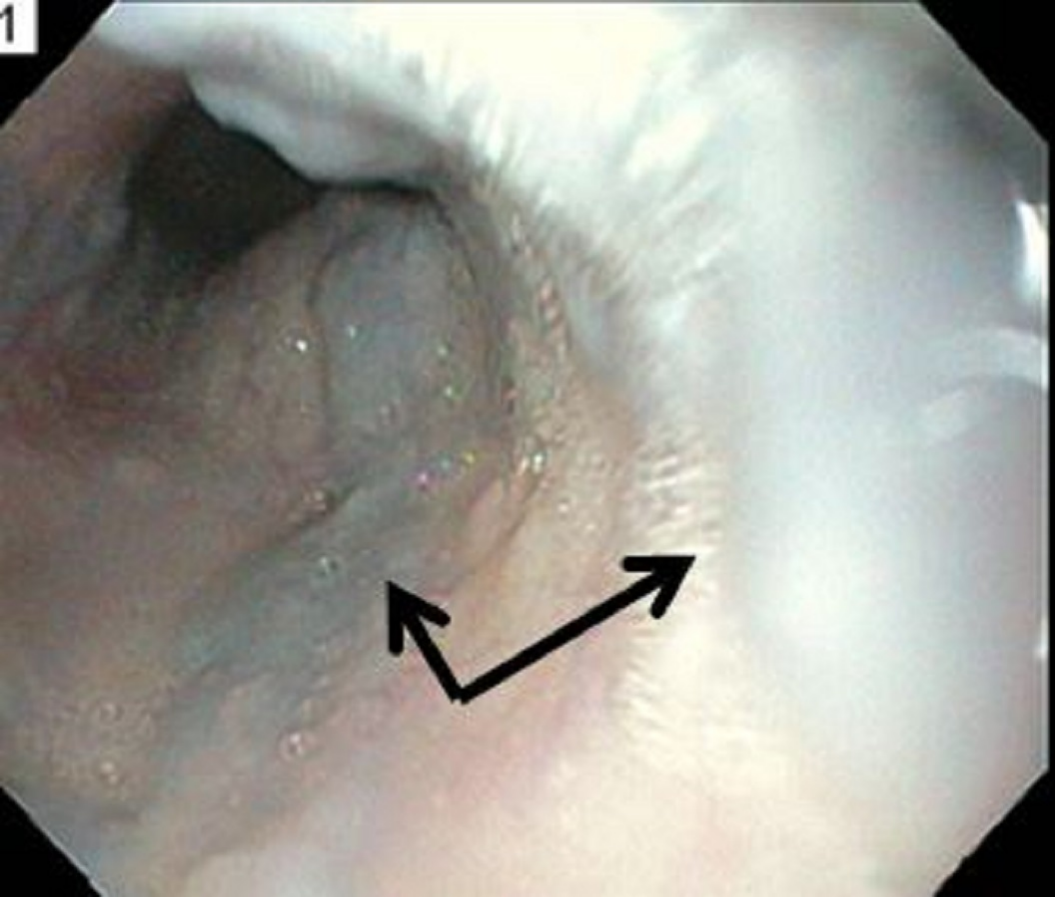
Esophagoscopy showing moderately sized mid-esophageal varices (arrows)

Intravenous lines were secured and the patient was started on intravenous proton pump inhibitors, an octreotide drip, and intravenous ceftriaxone for possible esophageal bleeding from cirrhosis in the emergency department. The patient was admitted to the intensive care unit for close monitoring. The patient underwent esophagogastroscopy after resuscitation, which revealed esophageal varices in the mid-esophageal area but no active bleeding was found (Figure 2). The varices were of moderate size with no red wale signs. Variceal band ligation was not performed.

**Figure 3:**
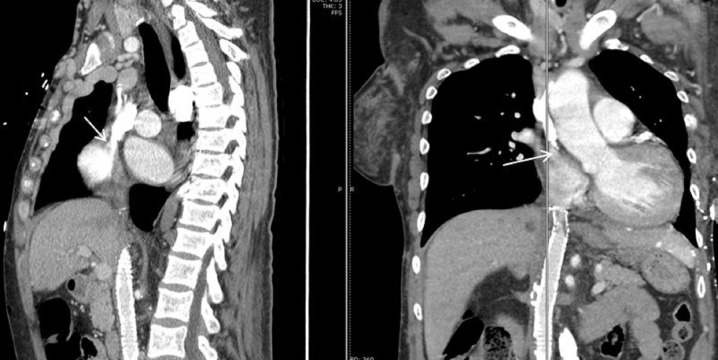
CT chest with contrast showing significant narrowing of the superior vena cava at the right atrial junction (white arrow)

**Figure 4:**
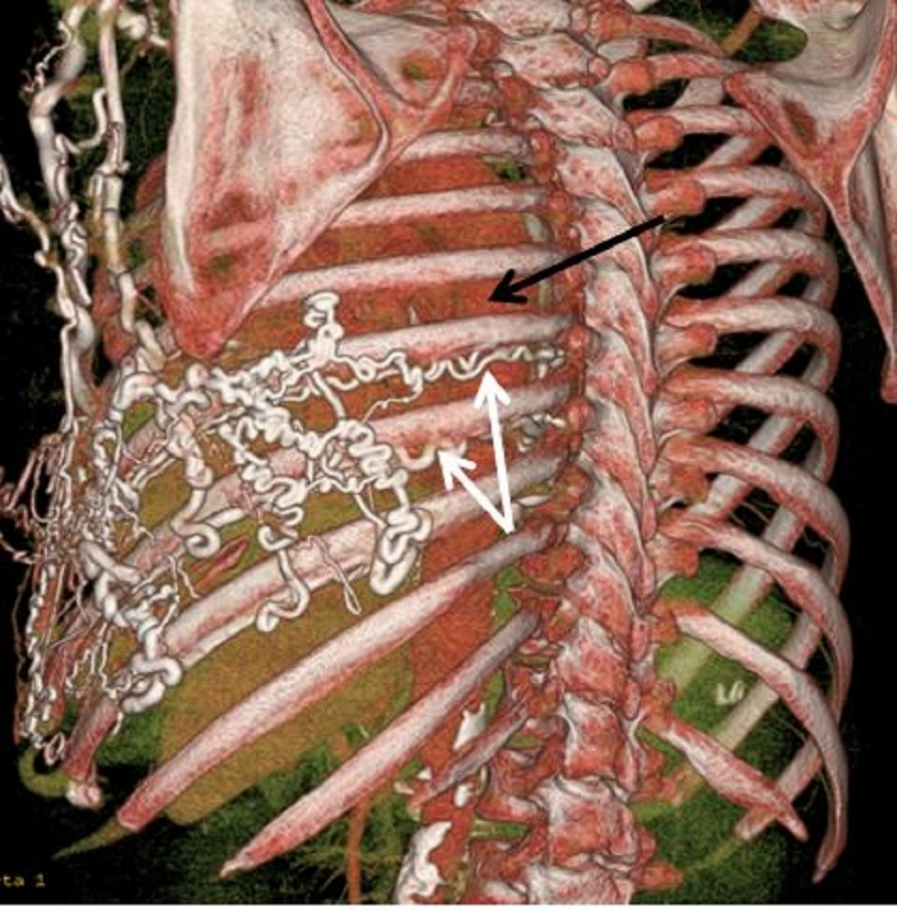
A three dimensional reconstruction of the CT scan showing marked engorgement of collateral vessels (white arrows) around the esophagus (black arrow)

An abdominal ultrasound did not show features of cirrhosis or portal hypertension. Computed tomography (CT) of the chest with intravenous contrast showed significant superior vena cava narrowing at the right atrial junction with large collateral venous channels, including a prominent right internal mammary vein and a large azygous vein (Figures 3, 4). Collateral vessels in the posterior left and anterior chest walls were also appreciated.

The patient was managed conservatively. The intravenous proton pump inhibitor, octreotide, and ceftriaxone were discontinued as the patient did not have signs of liver disease or peptic ulcer disease. She was evaluated by vascular surgery for a possible bypass surgery to the superior vena cava stricture area. She was discharged in stable condition with outpatient follow-up with the cardiovascular surgeon.

## Discussion

The most common cause (60% of cases) of SVC obstruction is extrinsic compression from a mediastinal malignancy, such as thymoma, lymphoma, or lung cancer [3]. Superior vena cava stenosis is also a well-documented complication of central venous dialysis catheters [4-8].

Blood from the upper two-thirds of the esophagus flows from multiple connections to superior vena cava and blood from the lower third drains through a left gastric vein into the portal venous system. With SVC obstruction, upper body venous drainage returns blood to the right atrium via collateral pathways, including cephalocaudal diversion of flow through the venous plexus of the esophagus. Such retrograde plexus flow produces “downhill varices” [4]. Reduced bleeding risk compared to uphill varices is due to the anatomic location. Typically, uphill varices are superficial and located near gastroesophageal junction, where they are easily eroded by reflux acid [5-6]. In contrast, variceal formations secondary to SVC are mostly submucosal and are afforded greater protection.

In most patients, upper gastrointestinal (GI) bleeding is the initial presentation. Physical examination will show engorged veins as shown in our patient (Figure 1). Direct venography is the gold standard for the diagnosis of SVC obstruction. Computed tomography (CT) angiography may be helpful if the appropriate technique is used for contrast injection so that flow artifacts are minimized [9]. Figures 3 and 4 show CT angiography images of SVC obstruction and collaterals around the esophagus in our patient, respectively. Magnetic resonance venography (MRV) is another modality that can be used for direct visualization of veins. In most cases, the choice of modality depends on the availability of imaging and planned intervention.

Currently, there is limited data on the management of downhill varices. Options include open surgical bypass or percutaneous angioplasty with or without stent placement [10].

In some instances, SVC obstruction is recurrent or refractory, or the patient may refuse surgical intervention. In these cases, management is usually supportive. Upper GI endoscopy can be done to control variceal bleed with banding or sclerotherapy.

## Conclusions

This case represents a rare cause of acute upper gastrointestinal bleeding in an individual with a central line placed for dialysis leading to SVC thrombosis. Gastroenterologists must show a high index of suspicion and awareness of the patient's overall clinical condition in dealing with acute upper gastrointestinal bleeding in patients on hemodialysis or with a history of a central catheter, especially in patients with no stigmata of chronic liver disease. There are no standard guidelines to manage acute bleeding associated with ‘downhill’ varices; we suggest that improved knowledge, prompt diagnosis, and management on a case-by-case basis. Using available endoscopic, radiological, and surgical interventions may lead to successful outcomes.
